# Dating historical droughts from religious ceremonies, the international pro pluvia rogation database

**DOI:** 10.1038/s41597-021-00952-5

**Published:** 2021-07-20

**Authors:** Fernando Domínguez-Castro, María João Alcoforado, Nieves Bravo-Paredes, María Isabel Fernández-Fernández, Marcelo Fragoso, María Cruz Gallego, Ricardo García Herrera, Emmanuel Garnier, Gustavo Garza-Merodio, Ahmed M. El Kenawy, Borja Latorre, Iván Noguera, Dhais Peña-Angulo, Fergus Reig-Gracia, Luís Pedro Silva, José M. Vaquero, Sergio M. Vicente Serrano

**Affiliations:** 1 ARAID Foundation, Zaragoza, Spain; 2 Departamento de Geografía y Ordenación del Territorio, Universidad de Zaragoza, Zaragoza, Spain; 3 Centre for Geographical Studies, Institute of Geography and Spatial Planning, Universidade de Lisboa, Lisbon, Portugal; 4 Departamento de Física, Universidad de Extremadura, Badajoz, Spain; 5 Departamento de Física de la Tierra y Astrofísica, Universidad Complutense de Madrid, Madrid, Spain; 6 Instituto de Geociencias (IGEO), CSIC-UCM, Madrid, Spain; 7 UMR CNRS Chrono-Environnement, University of Bourgogne Franche-Comté, Besançon, France; 8 Instituto de Geografía, Universidad Nacional Autónoma de México, Ciudad de México, Mexico; 9 Department of Geography, Mansoura University, Mansoura, Egypt; 10 Department of Geography, Sultan Qaboos University, Al Khoud, Muscat, Oman; 11 Estación Experimental Aula Dei, Consejo Superior de Investigaciones Científicas (EEAD-CSIC), Zaragoza, Spain; 12 Instituto Pirenaico de Ecología, Consejo Superior de Investigaciones Científicas (IPE-CSIC), Zaragoza, Spain; 13 Transdisciplinary Research Centre «Culture, Space and Memory» (CITCEM), University of Porto, Porto, Portugal; 14 Departamento de Física, Centro Universitario de Mérida, Universidad de Extremadura, Mérida, Spain

**Keywords:** Natural hazards, Climate sciences

## Abstract

Climate proxy data are required for improved understanding of climate variability and change in the pre-instrumental period. We present the first international initiative to compile and share information on pro pluvia rogation ceremonies, which is a well-studied proxy of agricultural drought. Currently, the database has more than 3500 dates of celebration of rogation ceremonies, providing information for 153 locations across 11 countries spanning the period from 1333 to 1949. This product provides data for better understanding of the pre-instrumental drought variability, validating natural proxies and model simulations, and multi-proxy rainfall reconstructions, amongst other climatic exercises. The database is freely available and can be easily accessed and visualized via http://inpro.unizar.es/.

## Abstract (summary)

**Table:** 

Measurement(s)	pro pluvia rogation date • proxy climate indicator
Technology Type(s)	digital curation
Sample Characteristic - Environment	drought
Sample Characteristic - Location	Mexico • Guatemala • Ecuador • Peru • Chile • Argentina • Portugal • Kingdom of Spain • French Republic • Italy • The Philippines

Machine-accessible metadata file describing the reported data: 10.6084/m9.figshare.14372093

## Background & Summary

Drought is one of the most important natural hazards, with adverse impacts on both natural and human environments^1^. These impacts span a wide variety of socioeconomic sectors, including agriculture^2,3^, energy^4^, and tourism^5^, among others. The environmental impacts of drought have also been well-documented^6^, as evidenced by forest decay and mortality^7^, forest fires^8^, changes in biodiversity^9^, etc. In the literature, much effort has been made to quantify, by means of drought indices, the spatial and temporal variability and changes of drought during the last 50 or 100 years^10,11^. Nonetheless, the relatively short-period of these comprehensive assessments does not allow for capturing some key characteristics and processes, such as trends and variability of drought in the pre-industrial era or the atmospheric mechanisms controlling this variability at multidecadal or longer time scales.

The rogation ceremonies for rain are a highly accurate documentary proxy of the occurrence of past agricultural droughts^12,13^. They were celebrated in different cultures and regions, in supplication to gods for changing the environmental or social risks brought to their communities. Although Catholics have been the most studied rogations with climatic perspectives, similar ceremonies have been found in many religions and societies^14^. A typical example in Islam is the *Salat al istisqa’*, in which *sunnah* is encouraged to pray for rain.

St. Mamertus, Bishop of Vienne, established the Roman Catholic rogations at the end of the fifth century^15^. The rogations had a well-defined bureaucratic process with letters and money transfer among institutions that have left paper records in various archives, primarily municipal and ecclesiastical, but also in archives of agricultural guilds, from which the date of the celebrations can be retrieved. The process of *pro pluvia* rogations (to beg for rain, hereinafter PPR) began when farmers noticed a lack of rainfall for crop development or cattle feeding. The farmer guilds sent a formal request to the local government to celebrate a PPR. The local government accepted this request, especially when crop failure was plausible, forwarding the request to the ecclesiastic authorities. Then, the ecclesiastic council decided on celebrating (or not) the ceremony and defined which liturgical act was required. If the decision was positive, the ecclesiastic council responded to the local government with the date and the proposed liturgy. Finally, the local government announced the rogation celebration^16^. In normal conditions, the cost of the celebration was paid by the local government a few days after the celebration. However, delays in these payments were documented frequently, as the ecclesiastical authorities claimed payments to local government months after the celebration of the rogation.

PPRs have been used in the last decades to understand drought variability in the pre-instrumental period across different countries (e.g. Spain^17–21^, France^22,23^, Ecuador^24^, Mexico^25–27^, Italy^28^ and Portugal^29^): to generate precipitation^30^ or atmospheric circulation modes reconstructions (e.g. North Atlantic Oscillation (NAO)^31^, El Niño-Southern Oscillation (ENSO)^32^); to validate natural proxies^33,34^, or to understand the social and ecological impacts of droughts^35^. All these works concur that PPRs are an accurate drought proxy, with extraordinary date precision. However, the rescue of PPR dates is a highly time-consuming task, given that it requires reading from hundreds of thousands of pages to extract relevant information to construct a PPR series dating back to three or four centuries. A key challenge of constructing PPR data is the capability of cities, villages, or towns to preserve their historical documentation and accordingly build series without gaps. In general, continuous series can be obtained for the last 500 years; the series suffered from data discontinuity before 1500.

Due to the demonstrated utility of this proxy, as well as the significant effort made by many researchers to rescue rogation ceremonies, a specific international repository for this proxy may be of particular importance for the research community. This repository can assure data quality and the reuse of this invaluable climatic information for different applications. For this reason, this work presents the INternational *Pro pluvia* ROgation database (INPRO), which incorporates information from more than 3500 PPRs spanning 153 different locations across the globe. The availability of this dataset for the research community will increase the reuse of PPRs in different climatic or social studies. Moreover, this initiative is open to any contributions of new research and we encourage any researcher to share their PPRs through the IMPRO initiative.

## Methods

**Fig. 1:**
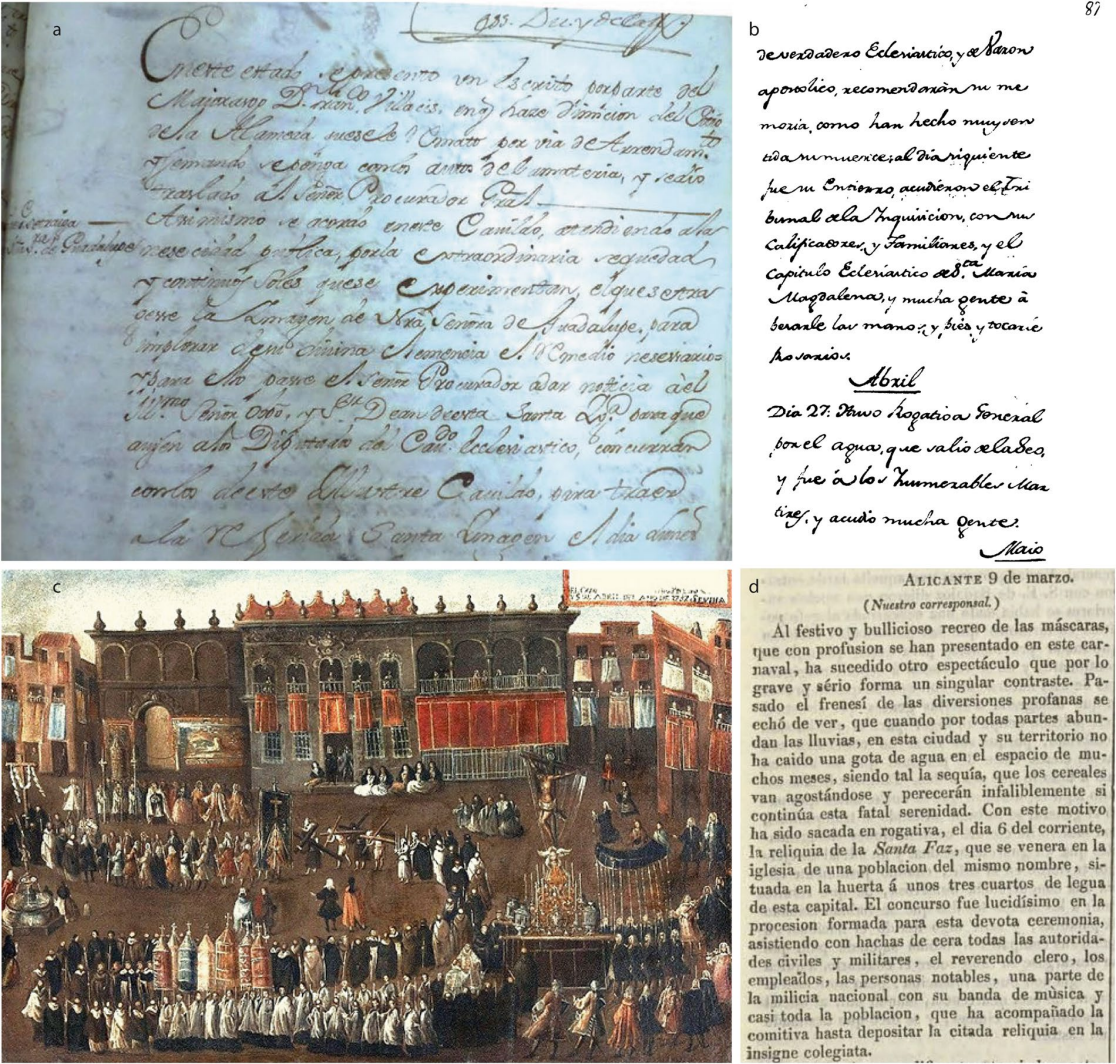
Examples of documentary sources: (**a**) Chapter act from Quito (5th February 1774), (**b**) Casa mayor’s private diary^40^ (27th April 1783), (**c**) Procesión de rogativa por falta de lluvias del Santo Crucifijo de San Agustín, ante el Ayuntamiento de Sevilla [Rogation procession by the lack of rainfalls of the Saint Agustine holy crucifix in front of Seville city council], ca. 1737, anonymous, oil on canvas^41^, and (**d**) *El Corresponsal* newspaper (12th March 1841).

A wide range of documentary sources can be used to retrieve information about rogations (Fig. 1). Each documentary source has different characteristics, especially in terms of consultation accessibility, time span, and dating precision. Here, we provide a brief description of the different types of documentary sources used. As illustrated, data were extracted from both primary and secondary sources. While primary sources refer mainly to those created when an event was reported from first-hand information, secondary sources describe events based on other sources. Herein, it is noteworthy to indicate that secondary sources must be handled with more care, given that they may contain errors originating from the transcription or interpretation of the documentary sources on which they are based. Nevertheless, the range of reliability of secondary sources is large, varying from secondary sources directly based on primary sources correctly cited to secondary sources that do not cite their sources and whose reliability is questionable.

### Primary sources

1. Official records of the institutions directly involved in the celebration of PPRs: Categorizes whether the identified virus is mosquito-borne or mosquito specific virus
  These records include ecclesiastical authorities, government institutions, and agricultural guilds. The bureaucratic process required to celebrate a rogation ceremony was documented in the official record books of these institutions, generating dossiers with the letters and payments crossed among different institutions. Unfortunately, the archives of the agricultural guilds are frequently lost. As such, we have focused only on governmental and ecclesiastical archives.
2. Official records books (chapter acts): They are the minutes of the assemblies of the local government or ecclesiastical authorities. Ordinary assemblies were normally celebrated on a weekly basis, but extraordinary assemblies were carried out more frequently to cope with emerging circumstances that required rapid reaction. Chapter acts reflect the decisions made by authorities over centuries. They have been frequently available in paper format. This increases the cost of consultation, and can make the task overwhelming, in some cases due to the limited opening hours of the archives preserving these acts. However, other collections have been digitized. These sources typically provide daily resolution, the motivations of the rogations are well defined, and the documentation can be complete and continuous for the last 400 or 500 years.
3. Rogation dossiers: they are preserved in ecclesiastical and local authorities archives. They contain the original letters that these authorities exchanged about the celebration of rogation ceremonies, from the request of agricultural corporations to payment of different services (e.g. candles, music). This documentation, when preserved, provides extraordinary detail about the development of liturgical acts, cost of ceremonies, and any discrepancy among ecclesiastical and municipal authorities. In some way, the official records described in the previous paragraph are a summary of these dossiers. Unfortunately, rogation dossiers are available only in a paper format, being mostly lost or uncatalogued. This makes their query a challenging task. The preserved dossiers were generated mainly from PPRs corresponding to extraordinary liturgical acts.
4. Private diaries: As PPR celebrations were rare and important events that disturbed ordinary life, the precise dates of some of these ceremonies were recorded in private diaries. Nevertheless, these diaries usually cover a short period of time (few decades) and miss some minor rogations.
5. Newspapers: Newspapers inform about important events that occurred in their area of interest. Rogation ceremonies were recorded frequently with their date, cause, and liturgical acts. Newspapers occasionally published rogations after the celebration, while on other occasions they published celebration announcements several days in advance. Indeed, this documentary source is not useful for generating continuous records at a specific location. Rather, it is more important for securing records in small towns or villages where other primary sources were lost. Newspapers were issued daily or weekly, first handwritten and printed from the 17^th^ century onwards. In the last decades, significant efforts have been made to digitize historical newspapers (e.g., https://prensahistorica.mcu.es, http://www.bne.es/es/Catalogos/HemerotecaDigital/, https://www.britishnewspaperarchive.co.uk, http://bndigital.bn.gov.br/hemeroteca-digital/, https://gallica.bnf.fr/). These historical sources allow for a digital and remote consultation of a great number of newspapers without the need of travelling to newspaper archives.
6. Iconographic testimonies: Engravings, paintings or photographs of pro pluvia rogations celebrations provide information about the ceremonies, such as participants, liturgy, streets decoration. Nevertheless, they are frequently restricted to celebrations with extraordinary liturgy.

### Secondary sources based on primary sources

In the last decades, some scientific works have provided information about PPRs. Commonly, these articles are based mainly on primary sources, which have been cited properly. Daily resolution is not always provided. This is the most reliable secondary source given that they were produced by scientists after careful data quality testing.

### Other secondary sources

Annals of cities, chronicles and similar books: References to rogation ceremonies can be found in books that recover the history of a city or a region. These are usually based on different sources that can be cited properly or not. This makes the traceability of rogations uncertain. The dates of rogations are not given on a daily basis in these sources. Rather, they often refer to these events at the monthly, seasonal, and annual scales. Importantly, only rogations associated with liturgical acts, such as processions or pilgrimages, are primarily documented.

Monographs about religious images or relics: these monographs provide information on the dates of the most important rogation ceremonies. However, dates of rogations are commonly provided at a coarser temporal scale. In addition, lists of rogations are mostly not exhaustive. Albeit with these shortcomings, these sources can provide valuable information about very old PPRs whose primary sources were lost.

For each document, we have compiled the date of PPR with the best available resolution. This is the most objective information and it is provided by all records with a resolution ranging from daily to annual. Other information used in some rogations indices (e.g. liturgical act^1614^, expenses^36^, area occupied by the text in chapter acts^37^) has not been compiled. This is simply because this information is not always available. Moreover, it can be more subjective and/or less comparable among different locations.

## Data Records

**Fig. 2:**
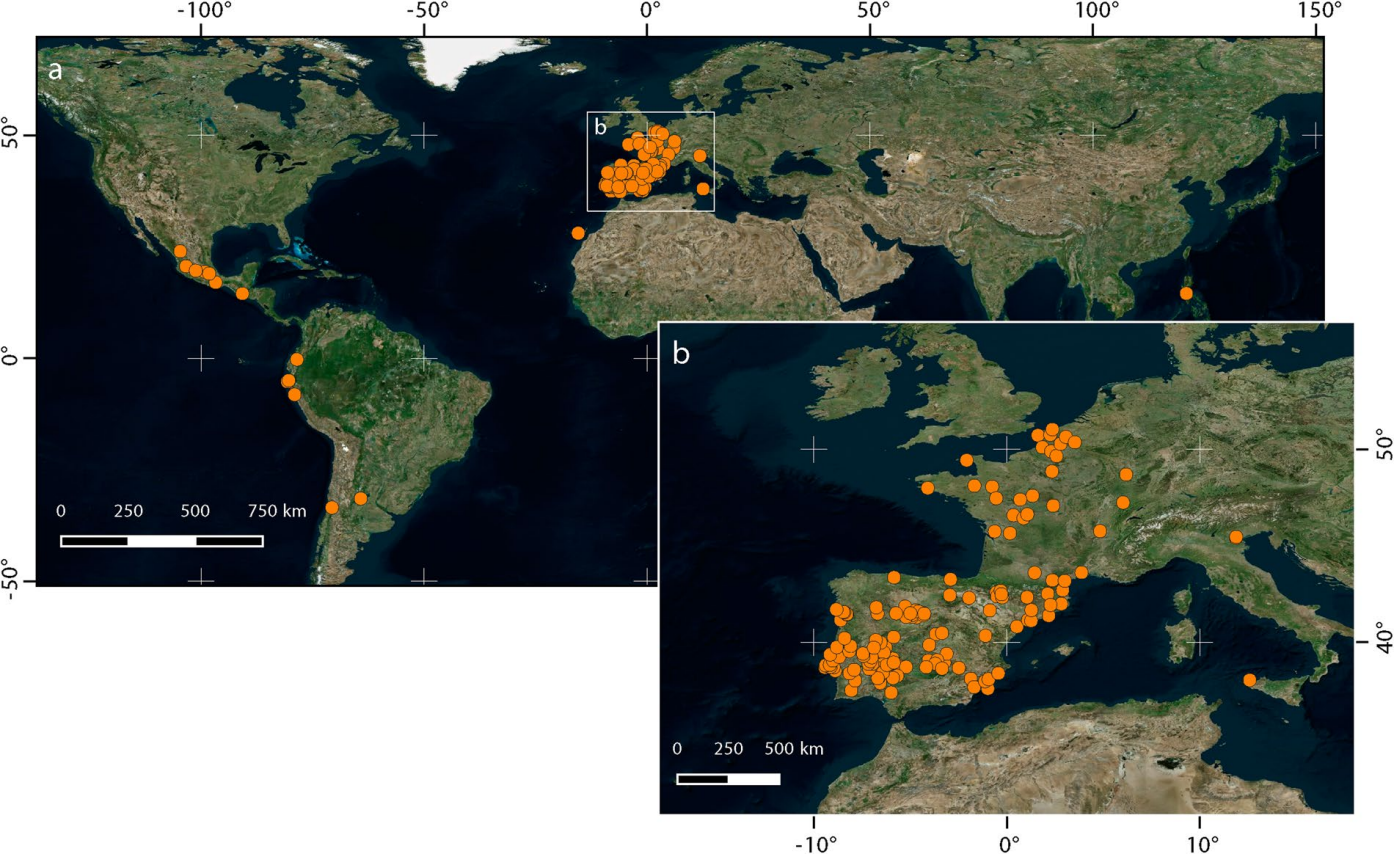
Locations of PPR series.

We retrieved 3536 dates of rogation ceremonies from 153 locations spanning 11 countries (Mexico, Guatemala, Ecuador, Peru, Chile, Argentina, Portugal, Spain, France, Italy, Philippines) (Fig. 2). The earliest records were collected from Spain, dating back to the 14^th^ century. The available records from France, Portugal, and Italy started in the 16^th^ century, while those located in Latin-America had data from the early 17^th^ century. The latest records are from the first half of the 20^th^ century, referring mainly to rural areas whose celebrations still have an important significance^38^.

The database is in figshare repository^39^. It is a unique .csv file. Each row provides information about the celebration of one PPR, including the date of the celebration, location (with the geographic coordinates), and documentary source from which it was retrieved.

Date of celebration: When possible, we provide the exact date of celebration of each rogation on a daily scale. The date recorded in the database is the date when PPR was celebrated. This is not a trivial question, because we had a bureaucratic period of around one week for each rogation (i.e. from the first time that famers requested celebration to the exact date of celebration). Some liturgical acts lasted some days (e.g. *novenas* (nine days) and *triduo* (three days)). In such cases, we provide the date of the first day. In our database, there are four columns related to the date: “year”, “season”, “month”, and “day”. We filled in these columns depending on the information provided by the document. We found that 48% of the rogation records had daily resolution, compared to 44% (seasonal) and 4% for monthly, and annual scales.

Location: We provide the name of the city or town where the PPR was celebrated and an approximate latitude and longitude in the World Geodetic System 1984 (WGS84). The coordinates were approximated to the center of the locations. When two or more rogations are held in the same location (e.g., cathedrals, churches, or monasteries), we treat them all as one location, with the same name and geographical coordinates.Type: All the retrieved PPR were referred to drought. We have flagged with an asterisk those celebrations in which the motive is not clearly specified in the document, but the researcher considers that it is caused by drought.

Documentary source: There are three fields to describe the documentary source: i) type of source (primary, secondary based on primary sources, and secondary); ii) details of the source from which data were retrieved; and iii) a reliability categorization of the source (see the technical validation section for details).

## Technical Validation

The validation of our dataset depends largely on the reliability of the documentary sources. For this reason, we calculated a reliability classification, with values ranging from low (1) to high (3) for each record:

1. For secondary sources that do not cite properly their primary documentary source or were directly based on secondary sources.
2. For secondary sources based on primary sources, which were cited correctly. These sources were produced by specialized researchers, with a possibility to verify the information from their primary source.
3. Only for primary sources with direct evidence.

Our findings reveal that 16%, 68%, and 16% of the PPRs had high, moderate, and low reliability, respectively. However, it should be noted that the level of reliability can be improved, following the consultation of new documents.

**Fig. 3:**
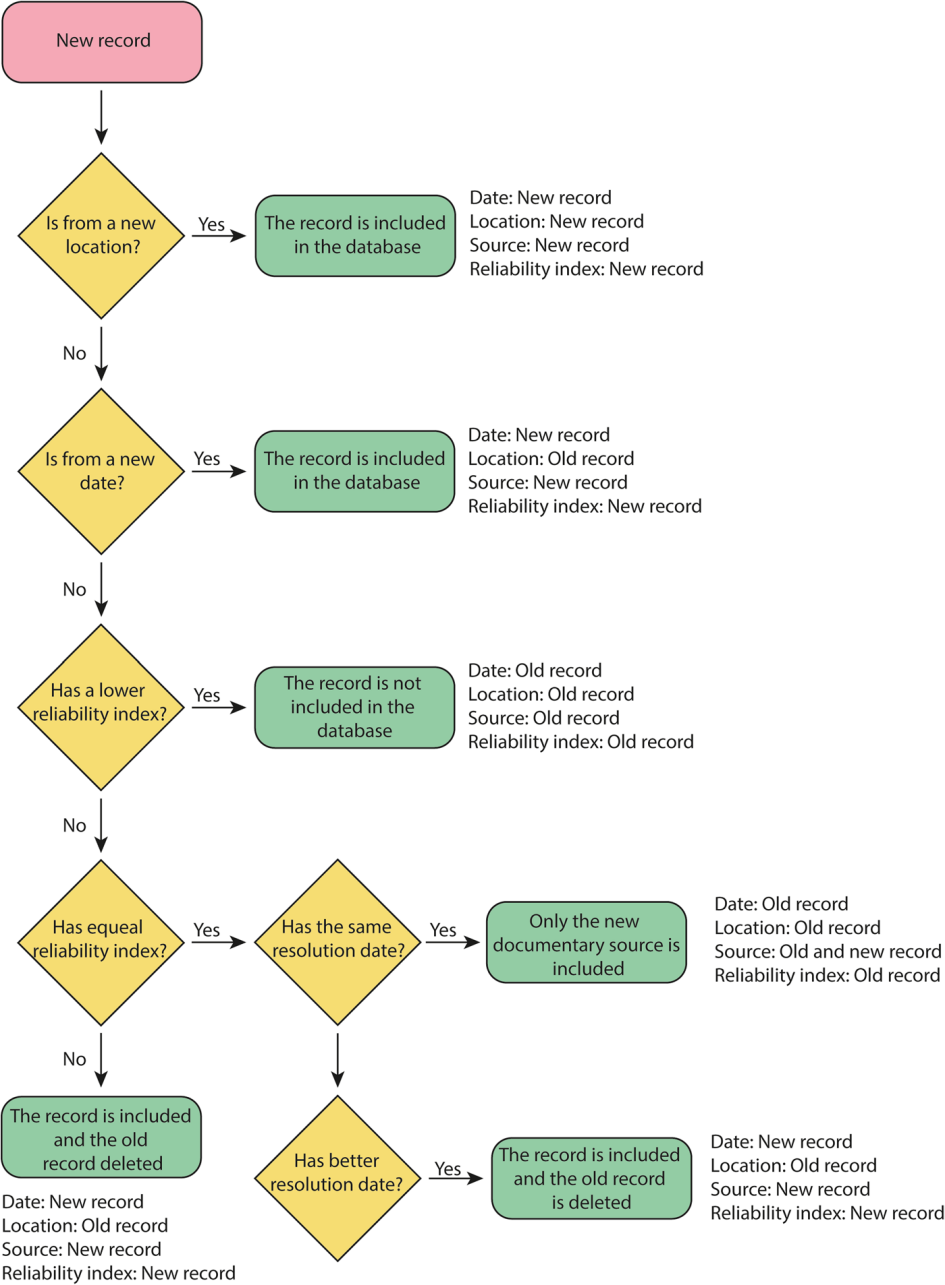
Flowchart for the inclusion of new records in the database.

Another important question in the quality of the dataset is record duplication. When the same PPR date was recorded in more than one source, the record with a higher value in the reliability index was retained, while the other was deleted. When both records had the same reliability index, we merged the records, providing details of both sources for the PPR record. Duplicated records were defined as two PPRs, belonging to the same location, with less than three days of difference. However, this definition extended also to identical dates provided at different temporal resolutions (e.g. a PPR recorded in 1798, winter 1798, February 1798 and 21st February of 1798). Figure 3 depicts a flowchart summarizing our procedure to assure data accuracy in terms of date, reliability, and location. This is a fundamental issue in database development, given that the frequency of celebration in a particular location during a period is used as an indicator of dryness during this period. This approximation cannot be used in PPRs database with duplications.

## Usage Notes

In addition to the figshare repository^39^ and for an easy consultation of the dataset, we have created a public web viewer, available at http://inpro.unizar.es/. This viewer allows direct inquiries for any time span between 1333 and 1949. For each location, we provided information about the total number of rogations for the selected period (centre of the circle) and the percentage of rogations in the different seasons: spring (MAM, green), summer (JJA, red), fall (SON, blue), winter (DJF, yellow), and no seasonal detail (black). When clicking on a location, information about the rogations for that location during the selected time period (such as location, rogation dates, and documentary sources) is displayed. Also, the complete database can be downloaded. The viewer is a useful tool for a quick screening of drought conditions in a particular location or period. The viewer is a useful tool for a quick screening of drought conditions in a particular location or period.

It is important to note that the PPRs are proxies of agricultural droughts and are thus associated with a loss of humidity in soils, which is controlled largely by a lack of precipitation^3,6^. As such, PPRs had a noticeable seasonality, associated with the requirements of soil humidity of the major crops or pastures dominating in each region for a particular time. This notion must be carefully taken into account when comparing information among regions or seasons. This makes the use of this proxy data more complicated on the annual scale.

The quality and homogeneity of the rescued series depend largely on the consulted documentary sources. Accordingly, long, continuous, and reliable series of rogation ceremonies can be very useful to promote our understanding of drought variability in a particular location and for individual dates. Also, the short series of rogation ceremonies can be appropriate to understand the extension or intensity of drought events detected by long rogation series or natural proxies, or to identify extreme droughts.

## Data Availability

The code used to generate the INPRO viewer is available via: https://github.com/lcsc/inpro.
